# Supplementary material for: Association between oral microbiome and seven types of cancers in East Asian population: a two-sample Mendelian randomization analysis
Source: Front Mol Biosci. 2023 Nov 21;10:1327893. doi: 10.3389/fmolb.2023.1327893 (PMC10702768; doi:10.3389/fmolb.2023.1327893)
Supplement: Supplementary file 1 [file DataSheet1.ZIP › Supplementary Materials/MR plots for tongue/tongue═╝/Pancreatic cancer/╥╚╧┘░⌐_╔╕╤í╜ß╣√/pheno.3074_to_pancreatic cancer_leave_one_out.pdf]

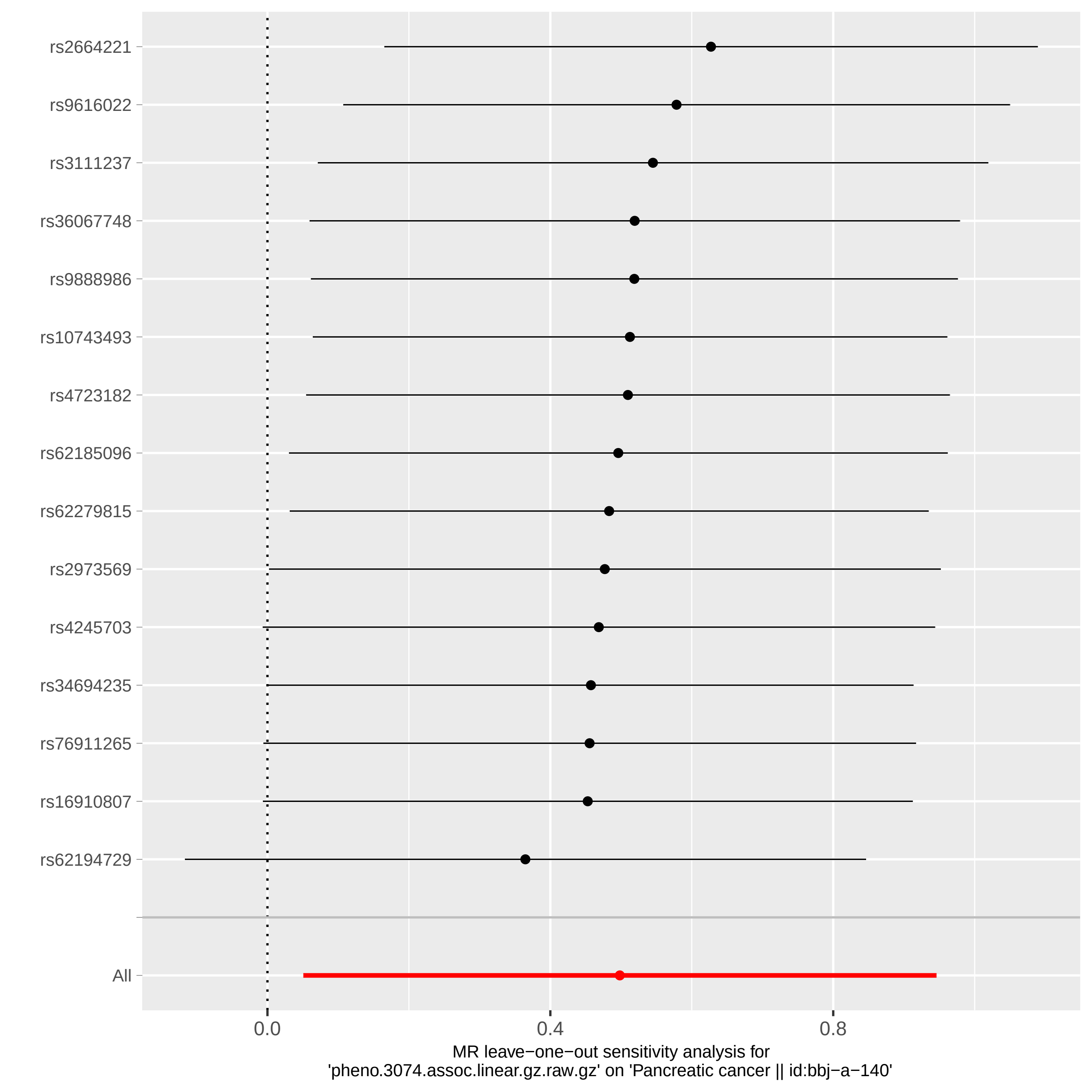
rs2664221
rs9616022
rs3111237
rs36067748
rs9888986
rs10743493
rs4723182
rs62185096
rs62279815
rs2973569
rs4245703
rs34694235
rs76911265
rs16910807
rs62194729
All
0.0
0.4
0.8
MR leave−one−out sensitivity analysis for
'pheno.3074.assoc.linear.gz.raw.gz' on 'Pancreatic cancer || id:bbj−a−140'
